# *AURKA* gene polymorphisms and central nervous system tumor susceptibility in Chinese children

**DOI:** 10.1007/s12672-021-00459-w

**Published:** 2021-12-15

**Authors:** Yong-Ping Chen, Li Yuan, Hui-Ran Lin, Xiao-Kai Huang, Ji-Chen Ruan, Zhen-Jian Zhuo

**Affiliations:** 1 Department of Pediatric Surgery, Guangzhou Institute of Pediatrics, Guangdong Provincial Key Laboratory of Research in Structural Birth Defect Disease, Guangzhou Women and Children’s Medical Center, Guangzhou Medical University, 9 Jinsui Road, Guangzhou, 510623 Guangdong China; 2 Department of Pathology, Guangzhou Women and Children’s Medical Center, Guangzhou Medical University, Guangzhou, 510623 Guangdong China; 3 Faculty of Medicine, Macau University of Science and Technology, Macau, 999078 China; 4 Department of Hematology, The Second Affiliated Hospital and Yuying Children’s Hospital of Wenzhou Medical University, 109 West Xueyuan Road, Wenzhou, 325027 Zhejiang China; 5 Laboratory Animal Center, School of Chemical Biology and Biotechnology, Peking University Shenzhen Graduate School, Shenzhen, 518055 Guangdong China

**Keywords:** *AURKA*, Chinese, CNS tumor, Polymorphism, Risk

## Abstract

**Introduction:**

Central nervous system (CNS) tumors comprise 15–20% of all malignancies occurring in childhood and adolescence. Previous researches have shown that overexpression and amplification of the *AURKA* gene could induce multiple human malignancies, with which the connection of CNS tumor susceptibility has not been extensively studied.

**Material and methods:**

In this study, we assessed whether and to what extent *AURKA* gene single nucleotide polymorphisms (SNPs) (rs1047972 C > T, rs2273535 T > A, rs8173 G > C) were associated with CNS tumor susceptibility, based on a case–control analysis in 191 CNS tumor patients and 248 controls. We determined this correlation using odds ratios (ORs) and 95% confidence intervals (CIs).

**Results:**

*AURKA* gene rs8173 G > C exhibited a crucial function to CNS tumor susceptibility fall-off (GC/CC vs. GG: adjusted OR = 0.68, 95% CI = 0.46–0.998, *P* = 0.049). In addition, the combined effect of lowering the risk of developing CNS tumors was more pronounced in carriers with 3 protective genotypes than others (adjusted OR = 0.55, 95% CI = 0.31–0.98, *P* = 0.044). Further stratification analysis illustrated that the existence of rs8173 GC/CC and three protective genotypes lowered CNS tumor risk in some subgroups.

**Conclusions:**

Our research suggested that the *AURKA* gene rs8173 G > C could significantly reduce CNS tumor susceptibility in Chinese children. More functional experiments are needed to explore the role of the *AURKA* gene rs8173 G > C.

**Supplementary Information:**

The online version contains supplementary material available at 10.1007/s12672-021-00459-w.

## Introduction

Central nervous system (CNS) tumors represent the most common solid malignancy in childhood [1, 2]. Glioma, thought to root in neural stem cells, glial progenitors, and astrocytes, constituted approximately 50% of pediatric tumors and 80% of malignancies among children aged 0–14 years [3, 4]. The most common histologic type of gliomas in children and adolescents was pilocytic astrocytoma, which accounted for 29.4% [5, 6]. However, in the population as a whole, the highest incidence of gliomas was glioblastoma (3.22 per 100,000 population), representing about 15% of total primary brain tumors and other CNS tumors, 48.3% of primary malignant cerebral tumors, and 57.3% of all gliomas [4].

Most patients with CNS tumors show more than one symptom during the disease. The first most frequent symptom of many patients with high-grade gliomas is a headache. The second most common symptom is vomiting, mainly accompanied by weakness, seizures, memory loss, gait disorder, and behavioral difficulties. Concerning low-grade gliomas in children, they usually present stunting and extreme emaciation [7, 8]. Seriously, CNS tumors can develop complications affecting patients’ quality of life like endocrinopathies, infections, venous thromboembolism, and intracerebral edema [9]. As the most representative CNS tumors, gliomas are divided into circumscribed gliomas (WHO grade I) and diffusely infiltrating gliomas (WHO grade II-IV). The former is benign and can be resected completely to achieve healing, while the latter is barely remedied by resection alone, requiring adjuvant therapy such as radio- and chemo-therapy. Additionally, most low-grade gliomas and almost all high-grade glioma are relapsed ultimately, even developing into higher-grade gliomas [10].

CNS tumors can harm patients’ health. The damage not only results from cancer itself but the influence of treatment. Patients may suffer functional defects in motor, communication, and neurocognitive skills according to the impact of tumorous location and size. Acute dysfunction is likely to occur after surgical removal. Radiotherapy and chemotherapy are associated with long-term impaired neurocognitive and various toxic reactions, respectively. Moreover, ill effects such as fatigue and nausea can come with drug therapy. Patients with CNS tumors also face a substantial negative impact on their psychology [11].

Antecedently, research reports have discussed that in addition to inherited hazard elements, the other hazard elements of CNS tumors might include several environmental exposures, such as ionizing radiation, allergies, atopic diseases, and mobile phones. It was remarkable that brain-ionizing irradiation was considered a well-defined risk factor for brain tumors [12–14]. Among the genetic risk factors, seven genetic variants have been identified that increased the risk of CNS tumors [15]. *TERT*, *MPHOSPH6*, *ACYP2*, and *ZNF208* genes have been identified to impact the prognosis of CNS tumors [16]. However, these genetic mutations are insufficient to clarify the pathogenesis of CNS tumors, and more genetic variants need to be identified.

AURKA (STK15/BTAK), a member of the Aurora families, encodes the mitotic serine/threonine kinase and affects cell-cycle regulation, particularly mitotic process [17]. It is an oncogene located on chromosome 20q13.2 [18]. During the S phase of the cell cycle, AURKA is situated on the centrosomes and takes part in cell division by regulating the formation and separation of mitotic spindles [19]. Therefore, once AURKA is overexpressed, disrupting the assembly of the mitotic checkpoint complex, and forming defective chromatid separation, brings about abnormal mitosis and tumorigenesis [20, 21]. Moreover, deficiency of the *AURKA* gene can lead to damage in cell mitosis, cytokinesis, and genome [22]. Consequently, it is assumed that the *AURKA* gene can be a potential target for anticancer therapies, and small molecule inhibitors of AURKA attract increasing attention [19, 23].

For the past few years, many studies have linked AURKA to human cancers. For example, AURKA has momentous functions in the progression of gastrointestinal cancer risk [24]. Moreover, AURKA, an independent factor, is regarded as a prognostic marker in colorectal adenocarcinoma [26]. Single nucleotide polymorphisms (SNPs) are caused by mutations in a single base, characterized by substituting one nucleotide for another, resulting in polymorphism at the locus with variations [27]. Many previous studies demonstrated that SNPs in mitotic checkpoint genes like *AURKA* could alter tumorigenesis susceptibility. *AURKA* gene single nucleotide polymorphisms (SNPs) may be a biomarker for hepatocellular carcinoma [25]. The polymorphism rs911160 in *AURKA* was identified to impact gastric cancer [28]. Furthermore, AURKA Ile31 polymorphism was associated with altered transcriptional levels among patients with prostates [29].

Given the importance of these three *AURKA* gene SNPs to altered cancer risk, and the correlation of these *AURKA* gene polymorphisms with CNS tumors has not been studied. Therefore, we chose these *AURKA* SNPs to evaluate for the first time whether these *AURKA* genetic polymorphisms are associated with CNS tumor risk and the relationship’s strength in Chinese children.

## Methods

### Sample selection

The cases and controls were frequency-matched for age, gender and ethnicity. They were enrolled between 2005 and 2019 from Guangzhou Women and Children’s Medical Center, the Second Affiliated Hospital, and Yuying Children's Hospital of Wenzhou Medical University. Every patient in the case group was confirmed with CNS tumors. The inclusion criteria for CNS tumors patients were biopsy verified or histologically confirmed glioma. The biopsy requires ample tumor tissue derived from the contrast-enhancement margin of the lesion instead of necrotic core and subsequently histological diagnose [30]. Histologically classification is served as the gold standard to determine CNS tumors. Briefly, CNS tumors are diagnosed histologically by WHO classification. Grade I CNS tumors grow slowly and have well-demarcated tumor cells. Grade II CNS tumors also develop slowly but usually exhibit brain-invasive growth, which cannot be resected entirely. Grade III CNS tumors are characterized by anaplasia histologically, showing rapid growth, particularly high cellularity, cellular pleomorphism, and elevated mitotic activity. Grade IV CNS tumors are glioblastomas, microscopically different from grade III CNS tumors, which display microvascular proliferation and necrosis [11]. The control group with no history of malignancy was derived from those undergoing conventional check-up during the same period. The frequency distribution of chosen variables between the case and controls was listed in Additional file 1: Table S1. The parents or guardians of all subjects offered valid informed consent before this research. Furthermore, we conducted this investigation under the approval of the Institutional Review Board of the Guangzhou Women and Children’s Medical Center (No. 2016021650) in Feb 2016 and The Second Affiliated Hospital and Yuying Children's Hospital of Wenzhou Medical University (No. LCKY2019-165) in May 2019.

### Polymorphism selection and genotyping

We selected single nucleotide polymorphisms based on the established criteria in the previous studies [31, 32]. In general, the underlying SNPs were chosen in the 5’-flanking region, 3’- and 5’- untranslated regions, and the exons of the *AURKA* gene. Finally, we decided rs1047972 C > T, rs2273535 T > A, and rs8173 G > C in the *AURKA* gene to investigate their association with CNS tumor susceptibility in Chinese children. Using the TIANamp Blood DNA Kit (TianGen Biotech, Beijing, China), genomic DNA was isolated from the peripheral blood specimens of participants. As mentioned before [31, 32], the TaqMan real-time PCR was utilized in genotyping through a 7900 HT sequence detection system (Applied Biosystem, Foster City, CA).

### Statistical analysis

The differences between genotypes frequencies in the controls and Hardy–Weinberg equilibrium (HWE) were measured via goodness-of-fit χ^2^ test. The two-sided χ^2^ test was conducted to compare the discrepancies in the distributions of participants’ selected variables. Odds ratios (ORs) and 95% confidence intervals (CIs) were calculated using logistic regression analysis. Association of *AURKA* gene polymorphisms with CNS tumor risk was evaluated using ORs and 95% CIs adjusted for age and gender. *P*-value was less than 0.05, implying statistically significant. We performed the combined effect of genotypes using logistic regression analysis, subsequently evaluated the impact on CNS tumor risk by age-gender adjusted OR, 95% CI, and *P*-value. The statistical analysis was staged on SAS v10.0 (SAS Institute, Cary, NC, USA).

## Results

### Effect of AURKA gene SNPs on CNS tumor risk

The chosen *AURKA* SNPs in our research are all located on chromosome 20. The rs1047972 is localized in exon 3: 56, 386, 407, in which the nucleotide substitution usually exists between T and C. The rs2273535 is localized in exon 3: 56, 386, and its nucleotide substitution is A/C, A/G, and A/T. The rs8173 is localized in exon 9: 56, 369, 735, and the nucleotide substitution is C/G and C/T. 191 cases and 248 controls were successfully genotyped to investigate the impact of rs1047972, rs2273535, and rs8173 on CNS tumor risk in this study. All genotype frequency distributions of these SNPs conformed to Hardy–Weinberg equilibrium (HWE = 0.567 for rs1047972; HWE = 0.328 for rs2273535; HWE = 0.332 for rs8173). Table 1 included detailed results of these three SNPs’ genotype frequencies and their association with CNS tumor susceptibility. Of note, rs8173 G > C exhibited a prominently correlation with decreased CNS tumor risk under the dominant model (GC/CC vs. GG: adjusted OR = 0.68, 95% CI = 0.46–0.998, *P* = 0.049), which means the effect of GC/CC genotypes was more protective than GG genotype after the C allele was regarded as mutant type. No significant associations with CNS tumor risk were observed in the rest of SNPs (rs1047972 C > T, rs2273535 T > A). The combined effect of protective genotypes showed that participants with 3 protective genotypes had a 0.55-fold (adjusted OR = 0.55, 95% CI = 0.31–0.98, *P* = 0.044) reduction in the development of CNS tumors. Subjects with 1, 2 protective genotypes could not impact CNS tumor risk. A similar negative result was detected for 3 protective genotypes in comparison to 0–2 protective genotypes.

**Table 1:** Association between *AURKA* gene polymorphisms and CNS tumor susceptibility in Chinese children

Genotype	Cases (N = 191)	Controls (N = 248)	*P* ^a^	Crude OR (95% CI)	*P*	Adjusted OR (95% CI) ^b^	*P* ^b^
rs1047972 C > T (HWE = 0.567)							
CC	157 (82.20)	190 (76.61)		1.00		1.00	
CT	30 (15.71)	53 (21.37)		0.69 (0.42–1.12)	0.134	0.67 (0.41–1.10)	0.115
TT	4 (2.09)	5 (2.02)		0.97 (0.26–3.67)	0.962	0.93 (0.24–3.54)	0.910
Additive			0.221	0.77 (0.51–1.17)	0.222	0.76 (0.50–1.15)	0.188
Dominant	34 (17.80)	58 (23.39)	0.154	0.71 (0.44–1.14)	0.155	0.69 (0.43–1.12)	0.130
Recessive	187 (97.91)	243 (97.98)	0.954	1.04 (0.28–3.93)	0.954	1.00 (0.26–3.80)	0.997
rs2273535 T > A (HWE = 0.328)							
TT	88 (46.07)	105 (42.34)		1.00		1.00	
TA	87 (45.55)	118 (47.58)		0.88 (0.59–1.31)	0.526	0.90 (0.60–1.34)	0.589
AA	16 (8.38)	25 (10.08)		0.76 (0.38–1.52)	0.443	0.76 (0.38–1.53)	0.444
Additive			0.380	0.88 (0.65–1.18)	0.380	0.88 (0.66–1.19)	0.408
Dominant	103 (53.93)	143 (57.66)	0.435	0.86 (0.59–1.26)	0.435	0.87 (0.60–1.28)	0.484
Recessive	175 (91.62)	223 (89.92)	0.543	0.82 (0.42–1.58)	0.544	0.81 (0.42–1.57)	0.525
rs8173 G > C (HWE = 0.332)							
GG	85 (44.50)	87 (35.08)		1.00		1.00	
GC	86 (45.03)	124 (51.21)		0.69 (0.46–1.04)	0.076	0.70 (0.46–1.05)	0.082
CC	20 (10.47)	34 (13.71)		0.60 (0.32–1.13)	0.113	0.60 (0.32–1.13)	0.116
Additive			0.048	**0.75 (0.56–0.998)**	**0.049**	0.75 (0.56–1.00)	0.051
Dominant	106 (55.50)	161 (64.92)	0.045	**0.67 (0.46–0.99)**	**0.045**	**0.68 (0.46–0.998)**	**0.049**
Recessive	171 (89.53)	214 (86.29)	0.306	0.74 (0.41–1.33)	0.307	0.73 (0.41–1.33)	0.306
Combined effect of protective genotypes ^c^							
0	69 (36.13)	74 (29.84)	0.080	1.00		1.00	
1	27 (14.14)	36 (14.52)		0.80 (0.44–1.46)	0.475	0.78 (0.43–1.43)	0.424
2	69 (36.13)	88 (35.48)		0.84 (0.53–1.33)	0.455	0.85 (0.54–1.35)	0.490
3	26 (13.61)	50 (20.16)		**0.56 (0.31–0.99)**	**0.047**	**0.55 (0.31–0.98)**	**0.044**
0–2	165 (86.39)	198 (79.84)		1.00		1.00	
3	26 (13.61)	50 (20.16)	0.072	0.62 (0.37–1.05)	0.074	0.62 (0.37–1.04)	0.068

Bold indicates significant results with *P* < 0.05

*OR* odds ratio, *CI* confidence interval, *HWE* Hardy–Weinberg equilibrium

^a^χ^2^ test for genotype distributions between glioma patients and cancer-free controls

^b^Adjusted for age and gender

^c^Protective genotypes were carriers with rs1047972 CT/TT, rs2273535 TA/AA, rs8173 GC/CC genotypes

### Stratification analysis

Our stratified analysis discovered the relationship between rs8173 G > C polymorphism and CNS tumor susceptibility based on age, gender, subtypes, and clinical stage (Table 2). In comparison to GG genotype carriers, GC/CC genotype carriers were less susceptible to CNS tumors in males (adjusted OR = 0.47, 95% CI = 0.28–0.80, *P* = 0.005) and clinical stages I + II tumors (adjusted OR = 0.65, 95% CI = 0.43–0.99, *P* = 0.044). Meanwhile, in contrast to subjects with 0 to 2 protective genotypes, those with 3 protective genotypes had a 0.47-fold reduced risk of developing CNS tumors in males (adjusted OR = 0.47, 95%CI = 0.22–0.99, *P* = 0.046). However, there was no evidence of correlation with CNS tumors in age and tumor subtypes.

**Table 2:** Stratification analysis of risk genotypes with CNS tumor susceptibility

Variables	rs8173		AOR (95% CI) ^a^	*P* ^a^		Protective genotypes		AOR (95% CI) ^a^	*P* ^a^
	(cases/controls)					(cases/controls)			
	GG	GC/CC				0–2	3		
Age, month									
< 60	41/44	56/82	0.73 (0.42–1.26)	0.261		84/99	13/27	0.57 (0.27–1.17)	0.123
≥ 60	44/43	50/79	0.61 (0.35–1.06)	0.080		81/99	13/23	0.68 (0.33–1.44)	0.317
Gender									
Females	33/40	56/64	1.06 (0.59–1.92)		0.837	74/84	15/20	0.82 (0.39–1.73)	0.606
Males	52/47	50/97	**0.47 (0.28–0.80)**		**0.005**	91/114	11/30	**0.47 (0.22–0.99)**	**0.046**
Subtypes									
Astrocytic tumors	61/87	75/161	0.67 (0.43–1.03)		0.065	117/198	19/50	0.61 (0.34–1.09)	0.097
Ependymoma	14/87	19/161	0.70 (0.33–1.47)		0.345	29/198	4/50	0.55 (0.18–1.64)	0.282
Neuronal and mixed	6/87	8/161	0.67 (0.22–2.03)		0.483	12/198	2/50	0.66 (0.14–3.08)	0.598
Embryonal tumors	4/87	3/161	0.42 (0.08–2.16)		0.300	6/198	1/50	0.64 (0.06–6.40)	0.703
Clinical stages									
I	48/87	62/161	0.69 (0.43–1.09)		0.109	93/198	17/50	0.70 (0.38–1.29)	0.258
II	19/87	19/161	0.54 (0.27–1.07)		0.079	34/198	4/50	0.46 (0.16–1.36)	0.162
III	6/87	11/161	0.95 (0.34–2.68)		0.921	15/198	2/50	0.54 (0.12–2.45)	0.424
IV	12/87	13/161	0.66 (0.28–1.58)		0.354	22/198	3/50	0.50 (0.13–1.84)	0.294
I + II	67/87	81/161	**0.65 (0.43–0.99)**		**0.044**	127/198	21/50	0.65 (0.37–1.13)	0.128
III + IV	18/87	24/161	0.77 (0.39–1.51)		0.450	37/198	5/50	0.52 (0.19–1.41)	0.201

Bold indicates significant results with *P* < 0.05

*AOR* adjusted odds ratio, *CI* confidence interval

^a^Adjusted for age and gender, omitting the corresponding stratify factor

## Discussion

This case–control study was performed to analyze the relationship between *AURKA* gene SNPs and CNS tumor risk in 191 patients and 248 cancer-free controls.

Vast documents have indicated that AURKA ensured normal mitosis by maintaining centrosomes and spindle poles’ stability. If the *AURKA* gene was abnormally expressed, the process was defective, promoting the transformation and progression of several human malignancies. Thus, amplification and overexpression of AURKA were strongly associated with several tumor risks [33–35], particularly evident in certain gene polymorphisms. For example, Wang et al. indicated that genetic variations in *AURKA* played a predictive role in the early clinical status of hepatocellular carcinoma (HCC). The HCC development was more affected by *AURKA* gene rs1047972 (V57I) polymorphism (C > T) than other SNPs. The rs1047972 variant TT genotypes conferred higher HCC risk than CC genotypes [25]. Gastric cancer (GC) was influenced by *AURKA* rs1047972. The rs1047972 TT and CC + CT genotypes contributed to elevated GC development risk, especially intestinal, diffuse, and both types of GC, reported by Mesic et al. in 2016 [36]. The *AURKA* gene rs2273535 (T91A) comes from nucleotide position 91, boosting the transformation of I1e to Phe. The research by Lee et al. revealed that the *AURKA* rs2273535 variant was susceptible to oral cancer. Additionally, if this SNP were exposed to alcohol, betel quid, and cigarettes, it would combine with them, known as gene-environmental interaction, which could produce synergistic effects to increase oral cancer risk [37]. Furthermore, the correlation between *AURKA* overexpression and GC poor prognosis was identified by Zhou et al. The *AURKA* rs2273535 TT genotypes increased higher risk of GC tumorigenesis than AA genotypes [38]. These two polymorphisms (rs1047972 G > A, rs2273535 T > A) were detected in exon 3 of the *AURKA* gene. It has been studied that rs1047972 variants modified rs2273535 polymorphism function and protein secondary structure to reduce carcinogenesis risk. Of note, the protective effect of rs1047972 was obvious in Caucasians and breast cancer, while not among Asians [39]. In terms of literature on the *AURKA* gene rs8173 is rather lacking. Our previous research has reported that rs8173 G > C was conducive to decreasing children’s Wilms tumor susceptibility, especially individuals with rs8173 GC/CC genotypes in subgroups of > 18 months, male, and clinical stages III + IV [40]. Regrettably, the *AURKA* polymorphisms were not associated with neuroblastoma risk by another previous study [41]. Other *AURKA* SNPs such as rs2064863 and rs6024836 have been found to be related to cancer susceptibility [42, 43].

Thus, although the investigations of how these three *AURKA* SNPs impact tumor risk are still preliminary, the probability that these SNPs affect CNS tumors cannot be excluded. Given that *AURKA* SNPs are frequently observed in multiple human malignancies and the implication of *AURKA* SNPs in CNS tumors has not been investigated intensively. Therefore, we are the first to speculate that the susceptibility of CNS tumors may be affected by *AURKA* gene SNPs. Existing research demonstrated that Gli2/miR-124/AURKA axis might be the key to influencing AURKA’s effect on glioma. Gli2 is a glioma-associated oncogene transcription factor. The target gene of Gli2 can mediate the Hedgehog signaling pathway to facilitate the occurrence of glioma [44]. Briefly, miR-124 is suppressed because the Gli2 combines with the upstream region of miR-124 transcriptional initiation locus excessively, whereas miR-124 existence is relevant to AURKA expression, giving rise to the anomaly of AURKA expression in glioma cells, which ultimately promotes cell proliferation and tumorigenesis [45, 46]. The Gli2/miR-124/AURKA occupies an essential position during the growth and development of human glioma cells.

In this study, we have shed light on that *AURKA* rs8173 was associated with reduced CNS tumor risk. At the same time, there were no apparent correlations between other *AURKA* gene SNPs (rs1047972 and rs2273535) and CNS tumor susceptibility. Moreover, the combined effect of protective genotypes and stratification analysis suggested that only subjects with 3 protective genotypes reduced CNS tumor risk significantly. Besides, stratified analysis manifested carriers with *AURKA* gene rs8173 GC/CC were linked to decreased CNS tumor risk in the subgroup of male and clinical stages I + II diseases, with no findings in age and subtypes. In our study, although, *AURKA* rs1047972 revealed negative results for altering CNS tumor risk, *AURKA* rs1047972 variants were identified to change lung adenocarcinoma (LADC) development probably [47]. Moreover, the rs1047972 variants were associated with elevated gastric cancer (GC) risk and basal-like breast cancer [48, 49]. With no association of *AURKA* rs2273535 with CNS tumor susceptibility, *AURKA* rs2273535 polymorphisms were associated with overall enhanced cancer susceptibility, particularly breast cancer [50]. Furthermore, the SNP was a favorable prognostic factor for urothelial carcinoma patients treated with alisertib (an *AURKA* inhibitor) [51]. In a previous study, the correlation between *AURKA* rs8173 and breast cancer risk was reported by Hong Shi et al. [52]. In addition, the rs8173 G > C was associated with reduced Wilms tumor risk [40]. This study also indicated the critical role of *AURKA* rs8173 in cancer modification.

However, serval deficiencies in our study deserve to point out. In the first place, what we analyzed was not a large sample size but a relatively small sample size, only containing 191 patients and 248 controls, which was insufficient to produce a convincing statistic. For the sake of reducing fortuitous events to the utmost, more samples are needed for verification. In the second place, we only tested three *AURKA* SNPs in the research. More potentially functional CNS tumor risk-associated SNPs in the *AURKA* gene and other genes should be investigated later. Of note, our previous study indicated that *FTO* gene SNPs are unlikely to have significant effects on CNS tumor risk [53]. In that study, we also included the samples of Guangzhou and Wenzhou. More SNPs in diverse genes are needed to genotype. Finally, the case–control study conducted in this study might have been biased because the genotype distribution of the general population might not be represented by the genotype distribution studied in the hospital.

In this research, *AURKA* gene rs8173 G > C polymorphism was identified to confer decreased CNS tumor susceptibility significantly in Chinese children. Continuous studies with a larger sample size are warranted to elucidate the effect of *AURKA* gene SNPs on CNS tumor risk.

## Supplementary Information

**Additional file 1: Table S1**. Frequency distribution of selected variables in CNS tumor patients and cancer-free controls.

## Data Availability

All data generated or analyzed during this study are included in this published article and its supplementary information files.
