# Supplementary material for: AURKA gene polymorphisms and central nervous system tumor susceptibility in Chinese children
Source: Discov Oncol. 2021 Dec 15;12:62. doi: 10.1007/s12672-021-00459-w (PMC8777528; doi:10.1007/s12672-021-00459-w)
Supplement: Supplementary file 1 — Additional file 1: Table S1. Frequency distribution of selected variables in CNS tumor patients and cancer-free controls. [file 12672_2021_459_MOESM1_ESM.doc]

| **Table S1**. Frequency distribution of selected variables in CNS tumor patients and cancer-free controls | | | | | |
| --- | --- | --- | --- | --- | --- |
| Variables | Cases (N=191) | | Controls (N=248) | | *P^a^* |
|  | No. | % | No. | % |  |
| Age range, month | 2.60-168.00 | | 4.00-168.00 | | 0.997 |
| Mean ± SD | 62.74 ± 47.28 | | 53.90 ± 33.47 | |  |
| <60 | 97 | 50.79 | 126 | 50.81 |  |
| ≥60 | 94 | 49.21 | 122 | 49.19 |  |
| Gender |  |  |  |  | 0.329 |
| Female | 89 | 46.60 | 104 | 41.94 |  |
| Male | 102 | 53.40 | 144 | 58.06 |  |
| Subtypes | | | | | |
| Astrocytic tumors | 136 | 71.20 | / | / |  |
| Ependymoma | 33 | 17.28 | / | / |  |
| Neuronal and mixed neuronal-glial tumours | 14 | 7.33 | / | / |  |
| Embryonal tumors | 7 | 3.66 | / | / |  |
| NA | 1 | 0.52 | / | / |  |
| WHO stages | | | | | |
| I | 110 | 57.59 | / | / |  |
| II | 38 | 19.90 | / | / |  |
| III | 17 | 8.90 | / | / |  |
| IV | 25 | 13.09 | / | / |  |
| NA | 1 | 0.52 | / | / |  |
| SD, standard deviation; NA, not available.  ^a^ Two-sided χ^2^ test for distributions between glioma patients and cancer-free controls. | | | | | |
